# School composition, school culture and socioeconomic inequalities in young people's health: Multi‐level analysis of the Health Behaviour in School‐aged Children (HBSC) survey in Wales

**DOI:** 10.1002/berj.3265

**Published:** 2017-02-28

**Authors:** Graham F. Moore, Hannah J. Littlecott, Rhiannon Evans, Simon Murphy, Gillian Hewitt, Adam Fletcher

**Affiliations:** ^1^ Centre for the Development and Evaluation of Complex Interventions for Public Health Improvement (DECIPHer)School of Social SciencesCardiff UniversityUK

**Keywords:** school health, wellbeing, inequalities, young people

## Abstract

Health inequalities emerge during childhood and youth, before widening in adulthood. Theorising, testing and interrupting the mechanisms through which inequalities are perpetuated and sustained is vital. Schools are viewed as settings through which inequality in young people's health may be addressed, but few studies examine the social processes via which institutional structures reproduce or mitigate health inequalities. Informed by Markham and Aveyard's theory of human functioning and school organisation, including their concept of institutional boundaries, critical theories of marketisation and the concept of micro‐political practices within schools, this paper presents analysis of student survey data (*N *=* *9055) from 82 secondary schools in Wales. It examines the role of socioeconomic composition, social relationships at school and institutional priorities in mitigating or perpetuating health inequality. It finds that affluent schools were most unequal in terms of student health behaviours and subjective wellbeing. In relation to health behaviours, students from affluent families accrue a disproportionate benefit. For wellbeing, students from poorer families reported lower subjective wellbeing where attending more affluent schools. Student–staff relationships appear to be a key mechanism underpinning these effects: poor relationships with staff were predicted by a pupil's position within schools’ socioeconomic hierarchy and associated with worse health outcomes. That is, students from the poorest families reported better relationships with teachers where attending less affluent schools. Universal approaches engaging with these social processes are needed to reduce health inequalities.

## Background

Multifarious indicators of health, including disability‐free life years, self‐rated health, subjective wellbeing and life expectancy, are positively associated with socioeconomic status (SES) (Marmot *et al*., 2010). Youth is a critical period in the formation of protective and risk behaviours, while lower subjective wellbeing at this stage of the life course is associated with psychosocial problems in adulthood (Park, 2004). Social and economic patterning in these outcomes emerges during childhood (Hanson & Chen, 2007; Viner *et al*., 2012; Gammelgard *et al*., 2015; Moore & Littlecott, 2015), with inequalities at this stage in the life course currently widening in line with growing economic inequality (Elgar *et al*., 2015; Shackleton *et al*., 2016). Identifying and influencing the risk and protective factors associated with socioeconomic inequalities in young people's health is a UK (HM Government, 2010) and international policy priority (World Health Organization, 2014). Schools are commonly viewed as a key channel for the delivery of interventions to reduce childhood inequalities, in large part due to their capacity to reach whole populations (Moore *et al*., 2015). To date, however, the social and institutional processes through which health inequalities are perpetuated and maintained have received little attention. There is evidence that some school‐based actions reduce inequality, while others increase it (Moore *et al*., 2015), although the widespread tendency to ignore impact on inequality means that the extant evidence provides little insight into the actual or potential roles of schools in shaping health inequality.

Despite the dearth of research addressing health outcomes, research from the sociology of education has examined the influence of schools on socioeconomic patterning in educational outcomes. Evidence suggests independent associations of family and school‐level socioeconomic status with academic attainment (Caldas & Bankston, 1997; Marks *et al*., 2006); being from a more affluent family *and* attending a school with a higher proportion of more affluent students are independently associated with better educational performance. There is also evidence that school and family‐level socioeconomic status interact to produce differential educational outcomes. For example, the most socioeconomically disadvantaged students typically *benefit least* from attending socioeconomically advantaged schools, despite apparent exposure to the same resources and support structures (OECD, 2012). This perverse compositional outcome has been explained by a process termed the ‘frog‐pond’ effect (Marsh & Hau, 2003; Crosnoe, 2009; Okamoto *et al*., 2013). Within this process, the relatively small number of poorer students located towards the lower echelons of a school's social hierarchy amplifies the effects of socioeconomic inequality, such as adverse social comparison, stigmatisation, disengagement and psychosocial problems (Espenshade *et al*., 2005).

An earlier analysis of survey data collected from secondary schools in Wales in 2009/10 provided the first evidence of a frog‐pond effect for some health outcomes; lower school and family‐level socioeconomic status were independently associated with increased smoking, lower physical activity and fruit and vegetable consumption, with family affluence more strongly predicting better outcomes in more affluent schools (Moore & Littlecott, 2015). Utilising a multi‐level survey of secondary schools and students in Wales, the present study replicates and extends this analysis using data collected in 2013/14. Initially, we update and extend previous analyses, examining a wider range of health‐related outcomes, including cannabis use, self‐rated health and subjective wellbeing. Drawing on the concepts of ‘institutional boundaries’ (Bernstein, 1975; Markham & Aveyard, 2003) and ‘micro‐political practices’ (Benjamin, 2002; Ball, 2012), outlined below, we then test hypotheses about the mechanisms through which schools may mitigate or exacerbate health inequalities.

### The (re)production of inequalities, institutional boundaries and micro‐political practices

Within the domain of health research there is an emergent corpus of studies that have sought to progress understanding of the mechanisms through which schools may produce and reproduce health inequalities, both at a population and subgroup level (Bonell *et al*., 2013a). Markham and Aveyard's (2003) theory of human functioning and school organisation is the most comprehensive theory of how the school environment influences health (Bonell *et al*., 2013b). It resonates with qualitative research undertaken to understand young people's experience of schools (Jamal *et al*., 2013) and has informed new school health improvement interventions (e.g. Bonell *et al*., 2014). However, this theory, and the key concepts within it, have rarely been tested empirically other than through very crude proxy‐based ‘value‐added’ measures of attainment (Bonell *et al*., 2016).

The theory of human functioning and school organisation is underpinned by Aristotelian notions of human functioning; individuals are thought to only be in a position to *choose* positive health behaviours and outcomes when their capacity for practical reasoning (i.e. critically perceive reality and view problems and solutions from different perspectives) and affiliation (i.e. shared values and empathetic understanding of others’ orientations to meaning) are supported. Taken together, these capacities make the world an understandable place for individuals, where they may make informed decisions about their health, and are in possession of affiliations that offer the support, self‐esteem and confidence to execute these decisions.

Building on the work of Basil Bernstein, Markham and Aveyard (2003) situate the development of these human capacities within the developmental context of schools to theorise how different institutional environments can enable—or constrain—students in realising these potentials through: the instructional order (the means of developing knowledge and skills) and the regulatory order (the institutional norms, values and belief systems). When students are disengaged from the instructional order, detached from the regulatory order or alienated from both, they may fail to effectively engage with the learning environment and remain apathetic to the values of the school, which diminishes the opportunity to develop practical reasoning and affiliations.

However, as theorised by Bernstein (1975), students’ socioeconomic backgrounds influence their interactions with the instructional and regulatory order. Indeed, students from working‐class backgrounds may be more likely to adopt an ‘alienated’ response, rejecting the norms of their school due to incongruence with the values of their families and other aspects of life, whilst simultaneously being poorly equipped to effectively engage with the pedagogic transmission of the learning environment (Fletcher & Bonell, 2013). Paul Willis's (1977) classic ethnographic study of a school in the West Midlands provided rich, empirical evidence of these social and institutional processes in action: working‐class lads’ choices, such as ‘havin’ a laff’ rather than getting a qualification, were made in the context of, and in opposition to, highly constraining institutional features and teachers’ practices, which reflect and reproduce aspects of wider social and economic inequalities. Meanwhile, students from more middle‐class backgrounds typically demonstrate a more ‘committed’ response within a context which represents a more natural extension of the middle‐class home (Bernstein, 1975; Willis, 1977).

Postulated mechanisms to encourage commitment to the instructional and regulatory order, and improve health outcomes whilst reducing inequalities, include erosion of institutional boundaries between teachers and students and between peers (Markham & Aveyard, 2003; Bonell *et al*., 2016). For example, an erosion of boundaries between students and staff within a school may be achieved through increased student involvement in decision‐making and learning processes. Such involvement can offer insights for both students and staff into each other's realities, thus promoting greater awareness of multiple perspectives and strengthening students’ sense of being supported and valued. This can enhance the capacity for practical reasoning and affiliation, whilst drawing alienated students into the instructional and regulatory order. The relationships between students can also be shaped by these and other institutional instructional practices (e.g. banding/streaming) and regulatory approaches (e.g. use of restorative approaches), which shape boundaries and interactions among a student body.

A range of research over several decades has evidenced how strong educational cultures of inclusiveness and positive teacher–student relationships can account for significant and positive ‘school effects’ (Rutter, 1982). Multi‐level studies in the UK and the USA have found that more supportive school cultures, where students are most engaged, have better relationships with teachers and experience a more supportive environment, are associated with lower rates of smoking, drinking, drug use and violence (Bonell *et al*., 2013a), although these studies rely on proxy, ‘value‐added’ measures of the school environment derived from the residual of modelling educational attainment and student social profile. The quality of teacher–student interactions has also been found to play a significant role in shaping young people's emotional wellbeing (Kidger *et al*., 2009a,b; Suldo *et al*., 2009).

While schools from socioeconomically deprived areas frequently score lower on markers of educational quality, they often place greater emphasis on pastoral care and emotionally supportive relationships with students (Lupton, 2005). Although the theory of human functioning and school organisation does not delineate contextualised interactions explicitly, the Bernsteinian concepts of instructional and regulatory orders—and how young people can become disengaged, detached and alienated—suggest that young people attending poorer schools may experience more emotionally supportive relationships with teaching staff than they might where attending a school with a more affluent intake. Relationships with peers will also likely be impacted by relative social status. Within poorer schools, a dominant culture driven by norms associated with poverty may give rise to more uniform health risk. However, for socioeconomically disadvantaged students attending affluent schools, stigmatisation by the culturally dominant middle‐class students may lead to alienation from mainstream school values and the formation of subcultures that promote high‐risk health behaviours, such as smoking, and reproduce health inequalities (Fletcher & Bonell, 2013). However, while issues such as these have been explored qualitatively, no studies to date have systematically explored the extent to which students’ relationships with staff or peers, or their sense of being included in school decision‐making, are differentially experienced according to students’ relative socioeconomic position within their school.

Alongside the theory of human functioning and school organisation, public health researchers have also drawn on other bodies of theory and evidence from education to theorise how the marketisation of schools may impact on student health and health inequalities (Bonell *et al*., 2011). Educational sociologists in the UK have documented how marketised educational policies—which aim to promote choice and comparison between schools using inspection ratings, attainment metrics and ‘league tables’—have transformed school management and organisation (Gillborn & Youdell, 1999). These policies permeate the micro‐political practices of school managers and other staff motivated to improve their school's performance according to a narrow range of key performance indicators, including via ‘gaming’ attainment metrics (Ball, 2012). Marketisation thus drives forward an agenda of academic standards, commodifying achievement and inscribing hierarchical schemas based on grade performance (Benjamin, 2002).

Informed by this literature, it has been hypothesised that the micro‐politics of marketisation may shape how some school managers prioritise (or de‐prioritise) certain health‐related activities and/or groups of students, which may impact adversely on health outcomes (Bonell *et al*., 2011). Educational researchers have found that the use of quasi‐markets encourages school managers to de‐prioritise non‐academic activities, as well as groups of students who are least likely to contribute to improving a narrow range of educational ‘metrics’ (Gillborn & Youdell, 2001; Ball, 2012). Hence, for students from poorer backgrounds attending schools which solely emphasise academic credentials and place a lower priority on non‐academic issues such as health and wellbeing, it is perhaps likely that the effects of socioeconomic deprivation will be exacerbated. However, to date, no studies have examined the role of organisational commitment to health in moderating the effects of socioeconomic deprivation on student health.

### Aims

First, this paper replicates previous analyses with earlier data, which suggested that school and family‐level socioeconomic status are independently associated with poorer health behaviours, with a focus on additional outcomes of self‐rated health and subjective wellbeing (Moore & Littlecott, 2015). Second, it operationalises the concepts of ‘institutional boundaries’ (Bernstein, 1975; Markham & Aveyard, 2003) and ‘micro‐political practices’ (Benjamin, 2002) to test the mechanisms through which schools may impact upon student health and health inequalities. The concept of ‘institutional boundaries’ is operationalised through items examining students’ perceptions of their school social environment, which assessed relationships with teachers, peer relationships and involvement in class and school‐level decision‐making. The concept of ‘micro‐political practices’ is operationalised through a measurement of school manager's organisational commitment to student health, which assessed the extent to which schools prioritise educational performance compared to non‐academic outcomes, such as emotional or physical health.

The following hypotheses are tested in the analysis:

1. Students from low SES families experience less positive relationships with teachers and peers, and less involvement in school decision‐making (i.e. more rigid institutional boundaries) than their more affluent peers.
2. Socioeconomic gradients in these measures of institutional boundaries are stronger in schools with more affluent intakes.
3. Student health behaviours, self‐rated health and subjective wellbeing are positively associated with perceptions of relationships within the school social environment.
4. The interaction between school and family‐level SES in predicting health outcomes will be attenuated by perceptions of the school environment.
5. High levels of organisational commitment to health are associated with lower between and within‐school inequality in health‐related outcome measures.

## Methods

### Sampling, recruitment and data collection

This paper replicates and extends a previous analysis of the 2009/10 Health Behaviour in School‐aged Children (HBSC) survey (Moore & Littlecott, 2015), using the 2013/14 survey. The HBSC survey in Wales 2013/14 was a cross‐sectional study of young people aged 11–16 in a nationally representative sample of secondary schools in Wales. Wales is one of 43 countries participating in the HBSC study internationally (Roberts *et al*., 2009). In Wales, schools were also asked to complete questionnaires on the school environment and school health‐improvement actions (Moore *et al*., 2016). Maintained and independent secondary schools in Wales were stratified by local authority and eligibility for free school meals, then selected using probability proportionate to size (and with an element of disproportionate stratification to allow analysis at Local Health Board level). School head teachers were invited to take part in the survey by letter and followed up with telephone calls. Overall, 181 schools were invited to take part in order to reach the target sample size of 82 schools. Participating schools received £150 to cover any costs incurred due to participating. Ethical approval was obtained from the Cardiff University School of Social Sciences Research Ethics Committee.

Within each participating school (*N *=* *82), one mixed‐ability class (approximately 25 students) from each school year 7–11 was randomly selected by the school to participate. Data were collected between November 2013 and February 2014. Trained fieldworkers attended each data collection to ensure sufficient support and assistance where required. Teachers were present during data collection but remained at the front of the classroom so they could not see students’ responses. The school environment questionnaire was sent to head teachers, who were asked to nominate a member of staff to complete it. A school environment questionnaire was completed by a member of staff within 67 schools out of the 82 HBSC schools. The survey included questions regarding organisational structures for delivery of health improvement and the presence, breadth and depth of school health‐improvement activities. The questionnaire included items from school surveys in Canada (Cameron *et al*., 2007) and was tailored to include priority topics in the Welsh context. Schools were asked to answer in relation to years 7–11 (compulsory education years only). The following variables are used to assess: SES; students’ perceptions of the school social environment to assess staff–student and student–student relationships and institutional boundaries; student health outcomes; and schools’ organisational commitment to health.

#### Socioeconomic status

Welsh Government data on the percentage of students eligible for free school meals (FSM) (due to their parents receiving income support) within each secondary school is routinely available and FSM is included as a measure of school‐level SES for each HBSC school. The Family Affluence Scale (FAS; Currie *et al*., 2008) was used to capture family‐level SES for each student. This comprises measures of car and computer ownership, frequency of holidays and bedroom occupancy. For the 2013/14 HBSC survey, due to concerns that some items within FAS were losing their saliency as markers of affluence (due, for example, to the rapid proliferation of computer ownership), additional items were added relating to dishwasher ownership and the number of bathrooms in the home. Items were summed to form a measure of family affluence. Where aggregated at the school level, the original 4‐item FAS scale correlated strongly with FSM entitlement (*r *=* *0.67), although the full scale with added items demonstrated a stronger association (*r *=* *0.80). Hence, the new items were retained for the final variable.

#### Perceptions of school social environment

Three questions on a 5‐point Likert scale asked students to rate the extent to which they felt accepted by their teachers, that their teachers cared about them as a person and that they trusted their teachers. The items demonstrated good internal consistency and were summed to form a single measure of staff–student relationships (alpha = 0.80). Three further questions on a 5‐point Likert scale asked students whether they felt students in their class enjoyed being together, were kind and helpful and accepted them as they were. The items demonstrated good internal consistency and were summed to form a single measure (alpha = 0.69). To measure the level of perceived involvement in decision‐making, students were asked to indicate the extent to which students were involved in making class rules, selection of classroom tasks, how to work on tasks, organising school events, planning school projects and how seriously they felt their ideas were taken by the school. The six items loaded onto two separate factors [involvement in classroom decision‐making (alpha = 0.72) and involvement in school‐level decision‐making (alpha = 0.65)].

#### Health‐related outcome measures

Young people were asked how many days in the past week they had participated in 60 minutes of physical activity, with a response of <6 days coded 0 and six or seven days coded 1. Young people were asked how often they ate fruits and vegetables. A binary variable was created which classified young people as either consuming fruit and/or vegetables at least daily, or not. Smoking was assessed by asking young people how often they currently smoked, with response options of ‘I do not smoke’, ‘less than weekly’, ‘weekly’ or ‘daily’. A response of ‘I do not smoke’ was coded as non‐smoking, with all other responses coded as smoking. A single item asked young people how often they had drunk alcohol in the past 30 days. Any score other than ‘never’ or ‘1–2 times’ was considered regular drinking. A single item asked young people how often they had used cannabis in the past 30 days. Young people providing any response other than ‘never’ were considered cannabis users. As well as being analysed individually as dependent variables, these behavioural items (physical activity, fruit and vegetable consumption, smoking, drinking and cannabis use) were summed to form a ‘health behaviour index’ of 0 to 4, with 0 indicating the least healthy behavioural pattern and 4 the most. Students were asked to rate their general health on a scale of 1 (excellent) to 4 (poor), reversed prior to analysis so that a high score represented better self‐rated health. As a measure of subjective wellbeing, young people were asked to indicate, on a scale of 1 to 10, how satisfied they were with their life.

#### Organisational commitment to student health

On the school environment questionnaire, schools were asked to select up to four areas which had been prioritised by the senior management team in the past two academic years from a list of nine areas, including student emotional and mental health, student physical health and staff health, as well as items on educational performance and school environment. A score of 0 was assigned if neither student health item was selected, 1 if one was and 2 if both were. Schools were also asked if they had a written action plan for student health, and how often this was reviewed. A score of 0 was assigned if there was no action plan, 1 for action plans that were reviewed less than once a year and 2 if there was a written policy which was reviewed annually. These items were summed to form an ordinal scale scored from 0 (lowest level of organisational commitment to health) to 4 (highest level) of organisational commitment to health.

### Analysis

We began by replicating our previous analysis of 2009/10 HBSC data (Moore & Littlecott, 2015), constructing multi‐level logistic regression models (binary for individual health behaviours and ordinal for combined health behaviours, subjective wellbeing and self‐rated health), with students nested within schools. Individual‐level variables (FAS score, sex and age), school‐level FSM entitlement and a FAS*FSM cross‐level interaction term were included in all models. FAS and FSM variables were standardised to minimise multi‐collinearity. To test hypotheses 1 and 2, we then constructed models comprising these independent variables as predictors of young people's perceptions of their school environment. To test hypotheses 3 and 4, perceived school environment variables were added as independent variables. Finally, using data from the 67 schools for which a school environment questionnaire was completed, we added variables for organisational commitment to health and their interactions with FAS and FSM.

## Results

### Response rates and sample description

A total of 39 young people were withdrawn from the study by parents, 33 refused to participate and 772 were absent on the day of data collection, with data obtained from 9055 students (91.5%). Demographic variables are presented in Table 1. For all variables, responses were available for 95% or more of the 9055 students completing the survey.

**Table 1:** Sample description

	*N*	Mean (SD)/*N* (%)
Mean (SD) age	9010	13.7 (1.4)
% (*N*) female	9022	50.1 (4459)
Mean (SD) FAS (summed 6‐item scale)	8779	15.1 (2.3)
Mean (SD) FSM	9055	14.9 (8.4)
*N* (%) smokers	9029	465 (5.2)
*N* (%) frequent alcohol drinkers	8577	691 (8.1)
*N* (%) cannabis use in past month	8662	249 (2.9)
*N* (%) active >5 days	8931	2235 (25.0)
*N* (%) fruit and vegetable consumers	9030	4132 (45.8)
Mean (SD) self‐rated health	8892	3.1 (0.7)
Mean (SD) subjective wellbeing	8721	7.3 (1.9)

### Associations of school and family‐level affluence with health‐related outcome variables

In multi‐level models comprising family and school‐level markers of SES (Table 2), a higher level of family affluence was significantly associated with higher levels of healthy behaviour, self‐rated health and subjective wellbeing. For individual health behaviours, a trend towards better health behaviour with increased affluence was observed for all outcomes bar alcohol consumption, falling short of significance for cannabis use. School‐level affluence was associated with better health behaviour and an association with self‐rated health approached significance (*p *=* *0.05), although it did not independently predict subjective wellbeing. For individual health behaviours, a trend towards better health behaviour with increased school‐level affluence was observed for all outcomes, although it was significant only for fruit and vegetable consumption, cannabis use and smoking. There were significant FSM*FAS interactions for all variables except alcohol use. Hence, data are consistent with the hypothesis that family and school‐level socioeconomic status predict health outcomes independently from one another, while more affluent schools are more unequal in relation to health behaviour and subjective wellbeing.

**Table 2:** Odds ratios and 95% confidence intervals from multilevel logistic regression models testing associations of health and perceived school environment variables with school and family‐level socioeconomic status

	Smoking (*N *=* *8732)	Alcohol use (*N *=* *8306)	Cannabis (*N *=* *8390)	Fruit and vegetable (*N *=* *8738)	Physical activity (*N *=* *8641)	Health behaviour (*N *=* *7894)	Self‐rated health (*N *=* *8609)	Subjective wellbeing (*N *=* *8446)	Student–teacher relationship (*N *=* *8422)	Peer relationships (*N *=* *8527)	Student involvement in class decision‐making (*N *=* *8430)	Student involvement in school decision‐making (*N *=* *8419)
FAS	**0.88 (0.79 to 0.98)**	**1.13 (1.03 to 1.24)**	0.88 (0.75 to 1.04)	**1.28 (1.22 to 1.35)**	**1.29 (1.22 to 1.37)**	**1.29 (1.23 to 1.35)**	**1.30 (1.24 to 1.35)**	**1.27 (1.22 to 1.32)**	**1.09 (1.03 to 1.15)**	**1.12 (1.07 to 1.17)**	1.04 (0.99 to 1.09)	**1.06 (1.01 to 1.11)**
FSM	**1.15 (1.01 to 1.31)**	1.09 (0.95 to 1.25)	**1.28 (1.06 to 1.55)**	**0.71 (0.66 to 0.76)**	0.98 (0.92 to 1.05)	**0.78 (0.73 to 0.83)**	0.95 (0.90 to 1.00)	1.03 (0.96 to 1.11)	**1.25 (1.07 to 1.45)**	0.98 (0.90 to 1.06)	0.99 (0.90 to 1.09)	1.03 (0.96 to 1.12)
FAS*FSM	**1.25 (1.12 to 1.40)**	1.02 (0.93 to 1.12)	**1.21 (1.03 to 1.42)**	**0.93 (0.88 to 0.98)**	**0.93 (0.88 to 0.99)**	**0.90 (0.86 to 0.94)**	0.97 (0.93 to 1.01)	**0.95 (0.91 to 0.99)**	**0.92 (0.87 to 0.97)**	0.97 (0.93 to 1.02)	1.04 (0.99 to 1.09)	0.96 (0.92 to 1.01)
Gender	**1.38 (1.13 to 1.69)**	1.00 (0.84 to 1.18)	1.21 (0.92 to 1.60)	**1.30 (1.19 to 1.42)**	**0.53 (0.48 to 0.59)**	**0.89 (0.82 to 0.97)**	**0.61 (0.57 to 0.67)**	**0.78 (0.73 to 0.85)**	**0.89 (0.81 to 0.98)**	**0.83 (0.76 to 0.91)**	1.00 (0.90 to 1.10)	0.95 (0.87 to 1.04)
Age	**1.93 (1.78 to 2.10)**	2.11 (1.96 to 2.28)	**2.66 (2.31 to 3.06)**	**0.90 (0.87 to 0.92)**	**0.87 (0.83 to 0.90)**	**0.74 (0.72 to 0.76)**	**0.79 (0.77 to 0.82)**	**0.79 (0.77 to 0.81)**	**0.76 (0.73 to 0.79)**	**0.81 (0.79 to 0.84)**	**0.72 (0.70 to 0.75)**	**0.73 (0.70 to 0.75)**

Bold highlighting indicates statistical significance (i.e. *p* < 0.05).

**Figure 1:**
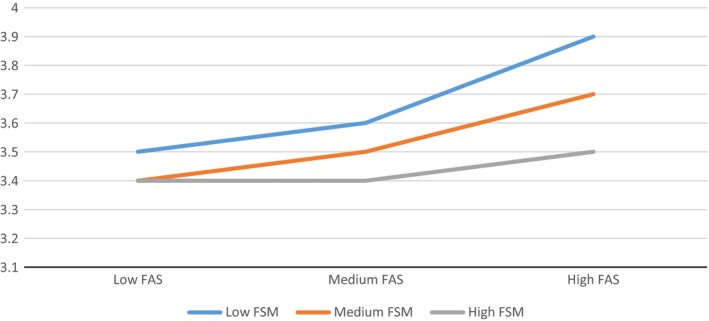
Associations between family affluence score and mean scores on the health behaviour index, by school type. [Colour figure can be viewed at wileyonlinelibrary.com]

To explore the interactions described above further, Figure 1 shows the relationship between family affluence and health behaviour in schools with low, medium and high FSM entitlement. Each line represents the association between FAS score and health behaviour separately for children attending low, medium and high FSM schools. Hence, inequality among pupils within school types is represented by the gap between the lines. Children from ‘low FAS’ families indicate similar levels of health behaviour (according to the combined index), regardless of school type. However, for ‘medium FAS’ students, there is a clear difference by school type indicated by the parting of the slope lines, with students attending more affluent schools reporting better health behaviours. This same trend, although *with further widening*, is evident for students from more affluent backgrounds. Hence, socioeconomic gradients in health behaviour are steepest in affluent schools, consistent with a hypothesis that the ‘benefits’ of attending a more affluent school are limited to students from more affluent families.

**Figure 2:**
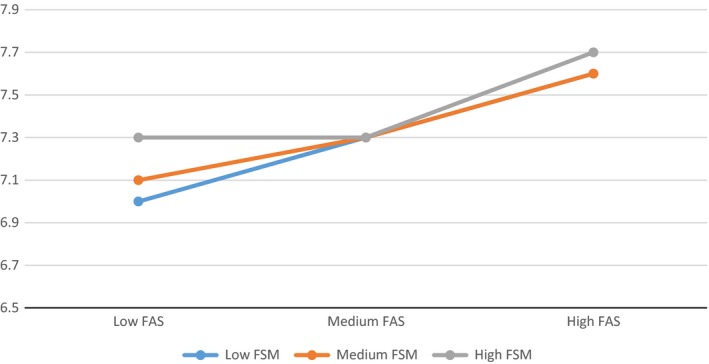
Associations between family affluence score and mean scores on a wellbeing scale, by school type. [Colour figure can be viewed at wileyonlinelibrary.com]

Figure 2 depicts the relationship between family affluence and subjective wellbeing in schools with low, medium and high FSM entitlement. For students from ‘low FAS’ (i.e. less affluent) families, mean wellbeing scores were highest where attending a school with a high FSM entitlement, and lowest where attending a more affluent school, as indicated by the discrepancy between scores on the left side of the graph. For students from more affluent families, there was less difference according to school type, as indicated by only a small difference between the values on the right side of the graph. Hence, as with health behaviour scores, there is evidence that the ‘effect’ of family affluence is greater in more affluent schools, as indicated by the relatively steep slope of the blue line. However, rather than a ‘benefit’ of attending a more affluent school which is limited to students from more affluent families (as with health behaviour), this is consistent with a hypothesis of an adverse effect on subjective wellbeing of attending more affluent schools for children from poorer families.

### Associations of school and family‐level affluence with perceptions of the school social environment

**Figure 3:**
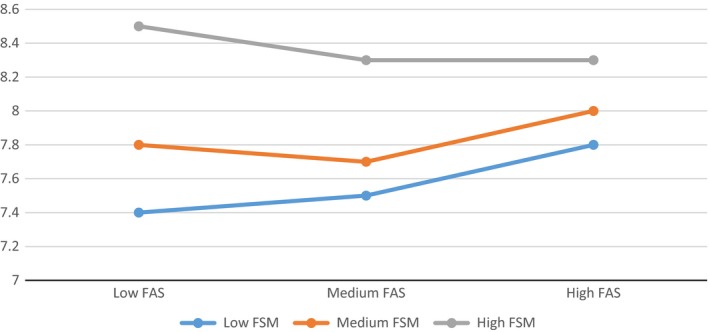
Associations between family affluence score and mean scores for perceived quality of relationships with teachers, by school type [Colour figure can be viewed at wileyonlinelibrary.com]

The odds ratios for variables relating to students’ perceptions of their school environment (Table 2) indicate that children from more affluent families were more likely to report positive relationships with teachers and peers, and were more likely to feel that they were involved in school decision‐making (with an association with class‐level decision‐making falling just short of significance). FSM entitlement was significantly associated with the quality of relationships with teachers, indicating that overall, a higher level of FSM entitlement was associated with *more* positive perceived relationships with teachers. For student–teacher relationships only, there was a significant FAS*FSM interaction, indicating that students from poorer backgrounds were less likely to perceive good relationships with their teachers where attending a more affluent school. This interaction is illustrated in Figure 3, which shows that the most positive student–teacher relationships were reported by poorer students attending more deprived (high FSM) schools, whereas the most negative relationships were reported by poorer children attending the most affluent schools. Notably, though, the discrepancy between school types is substantially smaller among students from more affluent families; even students from the most affluent families report better‐quality relationships with teachers where attending a poorer school.

### Associations of perceived social environment variables with health outcomes

Where models for health outcomes are rerun with the quality of student–teacher and peer relationships and perceptions of student involvement in decision‐making entered as independent variables (see Table 3), quality of student–teacher relationship emerges as the most consistent correlate of health outcomes. Better‐quality relationships with teachers are significantly associated with all individual health behaviours except physical activity, as well as improved self‐rated health and subjective wellbeing. Perceived quality of peer relationships is, by contrast, associated with physical activity, and fruit and vegetable consumption, though not substance use outcomes. Perceived quality of peer relationships is also significantly associated with both better self‐rated health and subjective wellbeing. Perceived student involvement in school‐level decision‐making is associated with better self‐rated health and subjective wellbeing, though not health behaviour, while involvement in class‐level decision‐making is associated only with self‐rated health. Notably, for substance use variables, particularly smoking and cannabis use, adjustment for perceived school environment variables substantially increases the strength of their association with FSM entitlement. An attenuated FAS*FSM interaction remains for some health behaviours, although it becomes non‐significant for subjective wellbeing after adjustment for school relationship variables.

**Table 3:** Odds ratios and 95% confidence intervals from multilevel logistic regression models testing associations of health with school and family‐level socioeconomic status and school environment variables

		Smoking	Alcohol use	Cannabis	Fruit and vegetable	Physical activity	Health behaviour	Self‐rated health	Subjective wellbeing
		(*N *=* *7922)	(*N *=* *7574)	(*N *=* *7628)	(*N *=* *7929)	(*N *=* *7853)	(*N *=* *7229)	(*N *=* *7888)	(*N *=* *7761)
All HBSC Wales schools (*N *=* *82)	FAS	0.93	**1.18**	0.92	**1.28**	**1.27**	**1.27**	**1.28**	**1.25**
		(0.83 to 1.04)	**(1.07 to 1.30)**	(0.78 to 1.09)	**(1.21 to 1.35)**	**(1.19 to 1.35)**	**(1.21 to 1.34)**	**(1.22 to 1.34)**	**(1.19 to 1.30)**
	FSM	**1.24**	1.13	**1.39**	**0.70**	0.99	**0.77**	**0.93**	1.00
		**(1.07 to 1.43)**	(0.97 to 1.31)	**(1.13 to 1.70)**	**(0.65 to 0.76)**	(0.92 to 1.05)	**(0.72 to 0.82)**	**(0.88 to 0.99)**	(0.94 to 1.06)
	FAS*FSM	**1.21**	0.99	1.13	**0.94**	**0.94**	**0.91**	0.98	0.97
		**(1.08 to 1.35)**	(0.90 to 1.10)	(0.96 to 1.34)	**(0.89 to 0.99)**	**(0.88 to 1.00)**	**(0.87 to 0.96)**	(0.94 to 1.02)	(0.93 to 1.01)
	Gender	**1.33**	0.92	1.05	**1.32**	**0.54**	0.92	**0.61**	**0.79**
		**(1.07 to 1.67)**	(0.77 to 1.10)	(0.78 to 1.42)	**(1.20 to 1.45)**	**(0.49 to 0.61)**	(0.84 to 1.01)	**(0.56 to 0.67)**	**(0.72 to 0.86)**
	Age	**1.81**	**2.03**	**2.54**	**0.92**	**0.86**	**0.77**	**0.86**	**0.88**
		**(1.71 to 2.08)**	**(1.87 to 2.20)**	**(2.16 to 2.97)**	**(0.89 to 0.95)**	**(0.83 to 0.90)**	**(0.75 to 0.80)**	**(0.83 to 0.89)**	**(0.86 to 0.91)**
	Student–teacher relationship quality	**0.84**	**0.88**	**0.78**	**1.04**	0.98	**1.07**	**1.08**	**1.14**
		**(0.81 to 0.88)**	**(0.84 to 0.91)**	**(0.74 to 0.83)**	**(1.02 to 1.06)**	(0.96 to 1.00)	**(1.05 to 1.09)**	**(1.06 to 1.10)**	**(1.12 to 1.16)**
	Peer relationship quality	0.97	1.01	1.00	**1.05**	**1.06**	**1.05**	**1.17**	**1.21**
		(0.92 to 1.02)	(0.96 to 1.05)	(0.93 to 1.07)	**(1.03 to 1.07)**	**(1.03 to 1.09)**	**(1.02 to 1.07)**	**(1.14 to 1.20)**	**(1.18 to 2.24)**
	Involvement in class decision‐making	**1.08**	1.02	1.02	0.99	1.01	1.00	**1.02**	1.00
		**(1.03 to 1.13)**	(0.98 to 1.06)	(0.96 to 1.09)	(0.97 to 1.01)	(0.98 to 1.03)	(0.98 to 1.02)	**(1.01 to 1.04)**	(0.98 to 1.02)
	Involvement in school decision‐making	0.95	0.97	1.01	1.01	0.99	1.01	**1.03**	**1.05**
		(0.91 to 1.00)	(0.93 to 1.01)	(0.94 to 1.09)	(0.98 to 1.03)	(0.97 to 1.02)	(0.99 to 1.03)	**(1.01 to 1.05)**	**(1.03 to 1.07)**
		*N *=* *6442	*N *=* *6153	*N *=* *6199	*N *=* *6444	*N *=* *6386	*N *=* *5876	*N *=* *6412	*N *=* *6299
All HBSC schools with SEQ data (*N *=* *67)	Organisational commitment to health	1.00	1.04	1.09	1.01	0.99	1.00	0.97	0.96
		(0.90 to 1.10)	(0.94 to 1.14)	(0.94 to 1.27)	(0.96 to 1.07)	(0.94 to 1.04)	(0.95 to 1.05)	(0.93 to 1.01)	(0.91 to 1.01)
	FAS*organisational commitment	1.08	1.01	1.08	0.97	1.00	0.97	1.00	**0.96**
		(0.99 to 1.17)	(0.94 to 1.09)	(0.95 to 1.23)	(0.93 to 1.01)	(0.95 to 1.05)	(0.93 to 1.01)	(0.97 to 1.04)	**(0.93 to 0.99)**

Bold highlighting indicates statistical significance (i.e. *p* < 0.05).

In models using data from the subset of students within schools where a school environment questionnaire (*N *=* *67) was completed, organisational commitment to health was not associated with health‐related outcome variables, neither was there a significant interaction between organisational commitment and FSM entitlement. There was, however, evidence of reduced within‐school inequality in terms of smoking (*p *=* *0.07) and subjective wellbeing (*p *=* *0.01) at schools with a higher commitment to health.

## Discussion

Consistent with previous analysis of 2009/10 HBSC data (Moore & Littlecott, 2015), this paper confirms that family and school‐level socioeconomic status are independently associated with a range of health behaviours among young people in Wales, and that more affluent schools are more unequal than poorer schools. The nature of this interaction is consistent with a conclusion that the apparent benefit of attending a more affluent school is experienced only by students from more affluent families; attending a more affluent school appears to make little difference to health behaviours of students from poorer families.

Extending this analysis to self‐rated health and subjective wellbeing revealed that both were predicted by family‐level SES, though not by school‐level SES. For subjective wellbeing there was also a significant interaction effect. However, by contrast to the interaction for health behaviour (which showed no effect of school type for children from poorer families, but a ‘benefit’ for those from more affluent families), this interaction was consistent with a conclusion that school type had little effect on subjective wellbeing for students from affluent families, but that attending a more affluent school led to lowered subjective wellbeing among young people from poorer families. Students from more affluent families consistently reported higher subjective wellbeing, varying little according to the school attended.

Our analysis goes beyond the descriptive analysis reported previously and also identifies mechanisms through which these between‐school differences in inequality might occur. Students from low SES families report less positive relationships with teachers and peers, and less involvement in school decision‐making. Only relationships with teachers, measured in terms of the extent to which students felt that they could trust their teacher, and that their teacher accepted and cared about them, varied significantly by school‐level affluence after adjusting for family affluence. This association was, however, in the inverse direction; students attending poorer schools reported significantly better relationships with their teachers. In particular, the interaction between school and family‐level SES suggested that students from the poorest families reported substantially better relationships with their teachers, if they attended a poorer school.

These findings are consistent with research on the frog‐pond effect (Marsh & Hau, 2003; Crosnoe, 2009; Okamoto *et al*., 2013), which suggests that the relative socioeconomic position of poorer students within a school's social hierarchy can lead students to feel undervalued by teachers and stigmatised. They are also consistent with educational studies which demonstrate a tendency for a greater emphasis on the provision of pastoral care and emotionally supportive relationships with students among schools with poorer intakes (Lupton, 2005). Within the educational literature, this tendency is often problematised, due to perceptions that diverting time towards pastoral support takes time away from educating students (Lupton, 2005). However, for the poorest students, this perhaps plays an important role in connecting them to their school and attenuating the health effects of disadvantage.

Consistent with Markham and Aveyard's (2003) theory of human functioning and school organisation, students’ perceptions of the extent to which they felt involved in school‐level decision‐making, as well as the quality of relationships with teachers and peers, were associated with better health outcomes. In particular, relationships with teachers predicted all health‐related outcomes bar physical activity, including a substantially lowered risk of substance use, improved self‐rated health and subjective wellbeing. The quality of relationships with peers predicted all outcomes other than substance use, while student involvement in school decision‐making predicted self‐rated health and subjective wellbeing. A study drawing on data collected from 40 schools in England in 2014 found associations between school and student‐level measures of lack of commitment and substance use (Bonell *et al*., 2016), which also provides evidence in support of Markham and Aveyard's theory.

Addition of perceived school environment variables to the model altered the associations of SES with health outcomes in two important ways. For substance use variables, the strength of association between school‐level SES and health behaviour substantially increased. This is consistent with a conclusion that inequality between schools in terms of substance use outcomes is lower than would be the case were it not for the higher perceived quality of student–teacher relationships in poorer schools. Second, again most notably for substance use variables and also for subjective wellbeing, the interaction between school and family‐level SES was reduced substantially by the inclusion of perceived school environment variables, consistent with a hypothesis that the greater inequality in more affluent schools is in part explained by the aforementioned differences in perceptions of the school environment. A tendency for marginalisation of poorer students within affluent schools, leading to the formation of subcultures whose identities are constructed around substance use, has been discussed in previous qualitative research (Fletcher & Bonell, 2013). However, these issues have not previously been examined quantitatively. Notably, included variables on perceptions of the school social environment made little difference to associations of SES with obesogenic behaviours, suggesting that within and between‐school inequalities are explained by mechanisms other than those tested in this study. This specificity of ‘school effects’ on substance use is both consistent with previous studies (West *et al*., 2004) and increases our confidence that this may be a causal relationship (Hill, 1965).

As reported elsewhere, organisational commitment to health is strongly correlated to the quantity of health‐improvement activity within secondary schools in Wales (Moore *et al*., 2016), particularly in relation to school health policy. However, there was no evidence from this study that a higher degree of organisational commitment to health predicted better health outcomes for students, nor was there any evidence of an ‘inverse care law’ (Hart, 1971), with no relationship between school affluence and the level of health services and activities provided. This suggests that marketised education policies and micro‐political management practices may not have the toxic effects on health previously suggested (Bonell *et al*., 2011), although these findings are specific to Wales and further research is required in other countries where marketisation is more extensive. These findings are, however, consistent with Markham and Aveyard's (2003) conceptualisation of school effects on health, which argues that it is the structure of ‘institutional boundaries’ and social relationships within schools which gives rise to better health outcomes among students and variations in outcomes between schools, rather than the delivery of specific health‐improvement actions. However, there was some evidence that a higher commitment to health reduced inequality in some outcomes, with a near significant interaction for smoking and a significant interaction for subjective wellbeing.

A key strength of this study is its large, representative sample of students and schools within Wales and the range of health items. However, the associations described are based on cross‐sectional data and, as such, no firm causal inferences can be made. Furthermore, the scope of data collection achieved in the HBSC survey is achieved at the sacrifice of some depth; measures of health behaviours and related outcomes are often based on single items with an unclear degree of measurement error. Nevertheless, the study has important, new theoretical and policy implications. Most notably, this study suggests that an emphasis on weakening boundaries between staff and students within schools may represent an important mechanism for improving health and reducing inequality.

While the public health literature is dominated by intervention approaches which focus on the installation of new packages of activities to address specific health topics, the social dynamics of schools and the social relationships within them may have the potential to influence a wide range of health‐related outcomes. There is some evidence that many poorer schools are already approaching the establishment of social relationships in a manner which may benefit student health and reduce inequalities. However, students from poorer backgrounds attending more affluent schools, where there is perhaps less perceived need for provision of pastoral support to offset the effects of socioeconomic disadvantage, may not receive such support and may be marginalised by a dominant middle‐class culture, with damaging health consequences.

To date, where impacts of schools on inequalities are debated, focus is placed predominantly on the pros and cons of targeting compared to universal approaches. There is growing support for attempting to address socioeconomic inequality via proportionate universalist approaches (Marmot *et al*., 2010), which is a midway position between universal and targeted approaches that propose intervention on a universal basis but with intensity varied according to need to address inequality more explicitly. The ‘pupil premium’ in England has provided additional funding linked to the proportion of children within a school entitled to receive free school meals. While intuitively attractive, the notion of being able to disproportionately reallocate health‐improvement activities in schools according to socioeconomic need in order to reduce inequalities in health is not unproblematic. It is largely premised on theories of health inequalities which focus on the (neo‐)material deficits in disadvantaged school environments as driving the health outcomes, and largely ignores theories and evidence that inequities are reproduced through social rather than purely material processes (Marmot, 2004; Wilkinson & Pickett, 2010).

## Conclusion

In conclusion, this study indicates that school and family‐level socioeconomic environments can, positively and adversely, influence young people's health. However, the ‘benefits’ of attending a more affluent school are not experienced equally throughout the socioeconomic distribution. Despite exposure to the same material resources, students from poorer families appear to gain little from attending a more affluent school in terms of improved health behaviour and experience negative consequences for subjective wellbeing. Emotionally supportive staff–student relationships are substantially predictive of a broad spectrum of health outcomes, while the quality of these relationships is predicted by a pupil's position within the socioeconomic hierarchy of their school. Proportionate universalist school health policies will have limited impact if they do not explicitly engage with social processes via which inequalities are reproduced within institutional contexts.
